# Parents’ understanding and attitudes toward the application of AI in pediatric healthcare: a cross-sectional survey study

**DOI:** 10.3389/fpubh.2025.1654482

**Published:** 2025-08-20

**Authors:** Yan-Dan Huang, Shu-Long Zeng, Jie Lin, Zhi-Peng Mao, Qi-Liang Zhang, Gao-Zhong Feng, Qiong-Xia Ou

**Affiliations:** ^1^Department of Pediatrics, Yongtai County Hospital, Fuzhou, China; ^2^Fujian Children’s Hospital (Fujian Branch of Shanghai Children’s Medical Center), College of Clinical Medicine for Obstetrics & Gynecology and Pediatrics, Fujian Medical University, Fuzhou, China

**Keywords:** AI, children, healthcare, understanding and attitudes, cross-sectional survey

## Abstract

**Objective:**

To investigate parents’ understanding and attitudes toward the application of Artificial Intelligence (AI) in pediatric healthcare.

**Methods:**

An observational, cross-sectional study was conducted using an *ad hoc* questionnaire. Between February and April 2025, 200 family members of children receiving care at our hospital voluntarily participated in the study. Inclusion criteria included being a family member of a child treated at the hospital. Exclusion criteria were an inability to understand the questionnaire and incomplete responses. The AI applications referenced by respondents primarily included large language models such as ChatGPT, DeepSeek, and Kimi, among others. The questionnaire consisted of two sections: demographic information, and attitudes toward the use of AI in pediatric healthcare. Data were analyzed using SPSS (version 25.0). Quantitative variables were expressed as mean and standard deviation, and categorical variables using frequencies and percentages. Group comparisons were performed using chi-square test and t-test (*p*-value < 0.05).

**Results:**

A total of 185 participants completed the questionnaire. Participants who were unaware of AI applications in pediatric healthcare were more likely to be older, have lower educational levels, and reside in rural areas. The majority of respondents (71.2%) believed that the information provided by AI was partially accurate, while 6.9% considered it partially inaccurate. Regarding perceived benefits, 74% identified convenience as the main advantage of AI in pediatric care, followed by 41.1% who cited high diagnostic efficiency. Key concerns included perceived inaccuracy and the potential for misdiagnosis (52%), as well as uncertainty regarding accountability in the event of an error (44.5%). Most participants (91.1%) believed that AI cannot replace doctors in the future.

**Conclusion:**

Although most parents were aware of the use of AI in pediatric healthcare and recognized its convenience and efficiency, they expressed concerns about accuracy, accountability, and data privacy. A notable lack of awareness was observed among older individuals, those with lower levels of education, and residents of rural areas.

## Introduction

Artificial Intelligence (AI) is a rapidly emerging computer engineering discipline that possesses human-like intelligence. In today’s world, AI has begun to transform multiple industries, including banking, healthcare, and travel, with its influence continually expanding (1). AI has brought unprecedented possibilities to healthcare. It is capable of supporting clinicians in performing a variety of core tasks and facilitating efficient communication (2). Additionally, the application of AI technology helps promote the accessibility of high-quality medical services, reduces the administrative burden on clinicians, optimizes the allocation and utilization of medical resources (3, 4). The research and application of AI are continuously expanding, and its integration with healthcare systems has become an important driving force in the development of medical technology (5). The use of AI based software is becoming increasingly common in all fields. Currently, AI technology has demonstrated superior performance in various areas, including clinical decision-making for pediatric diseases, image diagnosis in pathology, prediction of cardiovascular diseases, prognosis of cancer treatment, prediction of molecular changes in cancer, discovery and development of novel drugs, and control of epidemics (6–12).

When implementing any new technology in healthcare, it is crucial to consider the perspectives of patients (13). Insufficient acceptance of therapeutic measures can undermine patient compliance and worsen outcomes that might otherwise have been successful (14). Therefore, the rational use of AI in healthcare must also ensure its acceptability to patients and their families. However, many studies on the perception of AI in medicine have focused on healthcare professionals, none have specifically focused on parents’ understanding and attitudes toward the application of AI in pediatric healthcare. The aim of this study is to investigate parents’ understanding and attitudes toward the application of AI in pediatric healthcare and to provide recommendations for the further promotion of AI in this field.

## Methods

### Study design

This was an observational study with a cross-sectional survey. To our knowledge, no study has previously been reported on this topic. Therefore, we conducted this study as an exploratory study. This cross-sectional survey collected data using a *ad hoc* questionnaire. The ad hoc questionnaire was designed by the researchers.

### Setting

In this study, the AI of respondents used were referring primarily to the use of large language models such as ChatGPT, DeepSeek or Kimi.

### Calculation of study sample size

Based on the results of 78.9% of participants who were aware of the application of AI in pediatric healthcare, and assuming that the power of 0.85, the required number of participants was calculated to be 161. Assuming a 20% missing rate, the total sample size was set as 193.

### Participants

From February to April 2025, a total of 200 family members of children who visited our hospital voluntarily participated in this study. The 200 participants represented 200 unique family units (i.e., caregivers of 200 separate pediatric patients). A total of 185 completed the questionnaire. Inclusion criteria were: (1) family members of children treated in our hospital, (2) agreement to participate in this study. Exclusion criteria were: (1) inability to understand the content of the questionnaire, (2) incomplete questionnaire.

The survey was completed during the children visit to the hospital. The questionnaires were filled out individually under the supervision of the research team. If participants had any confusion regarding the questions, the researchers were available to promptly address their concerns or translate the questions into the local language to provide assistance. After completion of the questionnaires, the data were collected and analyzed separately by other researchers.

### Variables

The *ad hoc* questionnaire collected data in the following two aspects: (1) essential information regarding the demographic status of the participants (5 items) including: age, sex, education level, work and address; (2) attitudes toward the application of AI in pediatric healthcare (8 items).

### Statistical methods

SPSS 25.0 software was used for statistical analysis. The quantitative data were expressed as the mean ± standard deviation, the continuous data were in accordance with normal distribution by normal distribution test, *t*-test was used for statistical analysis. Categorical data were described using frequencies and percentages. Comparisons between groups were performed via the chi-square test. A *p*-value <0.05 was considered statistically significant.

### Ethical considerations

The present study was approved by Yongtai County Hospital, and adhered to the tenets of the Declaration of Helsinki. Prior to participation, the participants were informed of the purpose and content of the study. All participants were provided written informed consent and were informed that their participation was entirely voluntary and they had the right to withdraw for any reason.

## Results

A total of 200 family members of children participated in the study, of whom 185 completed the questionnaire. Table 1 showed the demographic characteristics of the participants. There were 53 males and 132 females, with age of 35.8 ± 9.2 years. The age distribution was as follows: 20–29 years (17.8%), 30–39 years (60.0%), 40–49 years (13.5%), and ≥50 years (8.7%). The majority of family members (78.9%) had completed high school or higher education, among whom 49.7% had an Bachelor’s degree, college diploma, or higher education level. Those with an educational level of junior high school or below accounted for 21.1%. Among the participants, 8.1% were white-collar workers or civil servants, 21.6% were professional and technical personnel, 2.1% were unemployed, 15.2% were farmers, and 53.0% had other occupations. A total of 62.7% of the participants resided in urban areas, while 37.3% lived in rural areas.

**Table 1:** Demographic characteristics of the participants.

Items	*n* = 185 (*n*%)
Age (year)	35.8±9.2
Distribution of age (year)	
20–29	33 (17.8%)
30–39	111 (60%)
40-49	25 (13.5%)
≥50	16 (8.7%)
Sex	
Male	53 (28.6%)
Female	132 (71.4%)
Education level	
Junior high school or below	39 (21.1%)
High school or secondary vocational school	54 (29.2%)
Bachelor’s degree or college diploma	87 (47%)
Master’s degree or above	5 (2.7%)
Work	
White-collar workers or civil servants	15 (8.1%)
Professional and technical personnel	40 (21.6%)
Unemployed individuals	4 (2.1%)
Farmers	28 (15.2%)
Other occupations	98 (53%)
Address	
Urban areas	116 (62.7%)
Rural areas	69 (37.3%)

The primary source of information on pediatric healthcare for the participants was the internet, accounting for 48.7% of the participants. AI was the main source of information for 18.4% of participants, while 28.6% participants primarily obtained information from healthcare professionals, and only 4.3% participants relied on books.

A total of 78.9% (*n* = 146) of participants were aware of the application of AI in pediatric healthcare, while the remaining 21.1% (*n* = 39) participants were not aware of the application of AI in pediatric healthcare. The age of participants who were unaware of the application of AI in pediatric healthcare was significantly older than those who were aware of its application (*p* < 0.05). The majority of participants who were unaware of the application of AI in healthcare had an educational level of junior high school or below and had significantly lower educational attainment than those who were aware of AI application (*p* = 0.001). Most participants who were unaware of the application of AI in healthcare resided in rural areas, which was significantly different from those who were aware of AI application (*p* = 0.001). Additionally, most participants who were unaware of the application of AI in healthcare were farmers, which was also significantly different from those who were aware of AI application (*p* = 0.001) (Table 2).

**Table 2:** Comparison of the demographic characteristics between participants who were aware of the application of AI in healthcare and those who were not aware.

Items	Participants were aware of the application of AI in healthcare *n* = 146	Participants were not aware of the application of AI in healthcare *n* = 39	*P*
Age (year)	34.4±7.2	38.0±11.5	0.009
Distribution of age (year)			
20–29	33 (17.8%)	1 (2.5%)	0.001
30–39	111 (60%)	9 (23.1%)	
40-49	25 (13.5%)	16 (41.1%)	
≥50	16 (8.7%)	13 (33.3%)	
Sex			
Male	53 (28.6%)	7 (17.9%)	0.170
Female	132 (71.4%)	32 (82.1%)	
Education level			
Junior high school or below	39 (21.1%)	30(76.9%)	0.001
High school or secondary vocational school	54 (29.2%)	9 (23.1%)	
Bachelor’s degree or college diploma	87 (47%)	0	
Master’s degree or above	5 (2.7%)	0	
Work			
White-collar workers or civil servants	15 (8.1%)	0	0.001
Professional and technical personnel	40 (21.6%)	0	
Unemployed individuals	4 (2.1%)	2 (5.1%)	
Farmers	28 (15.2%)	21 (53.8%)	
Other occupations	98 (53%)	16 (41.1%)	
Address			
Urban areas	116 (62.7%)	11 (28.2%)	0.001
Rural areas	69 (37.3%)	28 (71.8%)	

Among the 78.9% (*n* = 146) of participants who were aware of the application of AI in pediatric healthcare, 74% participants had used AI in pediatric healthcare. The majority (50.7%) of participants who had used AI in pediatric healthcare reported occasional use, 19.9% participants reported frequent use, and 3.4% participants reported always using AI. Most participants (71.2%) believed that the information provided by AI was partially accurate, while 6.9% participants thought that the information was partially inaccurate. The main advantages of using AI in pediatric healthcare, as perceived by the majority of participants, were convenience of use (74%) and high diagnostic efficiency (41.1%). The main disadvantages were perceived inaccuracy and the risk of misdiagnosis (52% participants) and unclear responsibility in case of misdiagnosis (44.5% participants). The vast majority participants (91.1%) believed that AI cannot replace doctors in the future (Table 3).

**Table 3:** Participants’ understanding and attitudes toward the application of AI in pediatric healthcare.

Items	*n* %
Q1. Did you aware of the application of AI in healthcare?	
Yes	146 (78.9%)
No	39 (21.1%)
Q2. Did you ever used AI in pediatric healthcare?	
Yes	108 (74%)
No	38 (26%)
Q3. How often do you use AI software when you encounter pediatric healthcare issues?	
Never	38 (26%)
Occasionally	74 (50.7%)
Often	29 (19.9%)
Always	5 (3.4%)
Q4. Do you think the pediatric healthcare information provided by AI is accurate?	
Very accurate	32 (21.9%)
Partly accurate	104 (71.2%)
Not accurate	10 (6.9%)
Q5. What do you think are the main advantages of using AI in pediatric healthcare? (Multiple choices allowed)	
Convenience of use	108 (74%)
Alleviation of medical resource tension	29 (19.8%)
High diagnostic efficiency	60 (41.1%)
Reduction of human error	10 (6.8%)
Q6. What do you think are the main disadvantages of using AI in pediatric healthcare? (Multiple choices allowed)	
Inaccuracy and the potential for misdiagnosis	76 (52%)
Lack of human touch	36 (24.6%)
Data privacy and security concerns	25 (17.1%)
Unclear attribution of responsibility in case of misdiagnosis	65 (44.5%)
Q7. Do you think that AI will be able to replace doctors in the future?	
Yes	13 (8.9%)
No	133 (91.1%)

## Discussion

The number of novel applications based on AI is steadily increasing and will be gradually implemented in clinical settings. Therefore, the use of AI-based technologies in healthcare is expected to grow rapidly in the coming years (15). Many AI tools have been widely accepted among healthcare professionals, thereby significantly improving various aspects of patient care and clinical workflow. The implementation of AI in healthcare holds great potential to transform patient outcomes and treatments (16). By leveraging AI-driven predictive analytics, clinical laboratory testing and disease detection can be made more accurate and efficient (17). AI can also assist in population health management by providing real-time relevant information and optimizing medication selection (18). The application of AI in digital health and mental health support has demonstrated its potential to enhance patient prognosis (19).

AI is also increasingly used in pediatrics. Bokov et al. (20) applied machine learning models to adjust the intervention measures for children with high-risk asthma, thereby improving the personalized care outcomes. Mumenin et al. (21) reported that machine learning can be used to predict the seizure status of newborns, thereby promoting early intervention and personalized care. In cardiology, Hunfeld et al. (22) employed machine learning models to identify congenital heart defects and predict outcomes following cardiac events. In gastroenterology, machine learning was used to manage chronic diseases such as Crohn’s disease and predict the risk of bacterial gastroenteritis in children (23, 24). Tong et al. (25) applied machine learning models to assess nutritional deficiencies and personalize dietary adjustments for children with chronic gastrointestinal diseases. However, to date, only a limited number of studies have reported professionals’ views of AI in healthcare. When implementing any new technology in healthcare, it is crucial to consider the perspectives of patients. There is currently a scarcity of research reporting patients’ views on the use of AI in healthcare (26). Moreover, no studies have been reported on parents’ understanding and attitudes toward the application of AI in pediatric healthcare. This study investigates and reports on parents’ understanding and attitudes toward the application of AI in pediatric healthcare. The AI that the families of the patients have come into contact with mainly refers to the use of large language models such as ChatGPT, DeepSeek or Kimi, etc.

In our study, the majority of participants (78.9%) were aware of the application of AI in healthcare, and a significant proportion (74%) among them had experience using AI in pediatric healthcare. Most individuals in this study believed that application of AI in pediatric healthcare offers the advantages of convenience and high diagnostic efficiency. These findings are similar to those of other studies. Aggarwal et al. (27) investigated the views of 408 participants in London and found that despite patients generally having limited prior knowledge of AI, the majority considered its use to be affirmative, trusted its application, and believed that the benefits outweighed the risks. Fritsch et al. (28) surveyed the attitudes and perceptions of 452 patients toward application of AI in healthcare and found that the majority were in favor of using AI in medicine and healthcare, with 53.18% of respondents rating it as positive or very favorable, and only 4.77% expressing negative or very negative opinions.

The integration of AI in healthcare has the potential to transform patient care, diagnostic procedures, and treatment methods. However, while the majority of people maintain a positive attitude toward the application of AI in healthcare, it still poses some challenges and limitations (29). First and foremost are the issues of information accuracy. The application of AI in medical diagnostics is still in its infancy, and given the complexity of various diseases and their symptoms, establishing accurate diagnostic techniques is highly challenging. In this study, 71.2% of participants believed that the information provided by AI for pediatric healthcare was only partially accurate. Furthermore, 52% of participants identified the main deficiency of AI in pediatric healthcare as insufficient diagnostic accuracy and the risk of misdiagnosis. Moreover, the vast majority of participants (91.1%) believed that even in a more advanced future, AI could never replace physicians. A study by Fritsch SJ et al. found that only a small proportion of patients would agree to be treated directly by AI, while the rest rejected AI treatment (28). In their study, all respondents believed that AI should not have too much autonomy and that physicians should closely monitor AI. They hoped that physicians should act as gatekeepers for AI decisions.

Secondly, the application of AI in healthcare also faces challenges related to ethical, privacy, and legal issues (30, 31). A review of AI in pediatric healthcare by Ganatra (32) reports that overcoming obstacles such as data limitations, ethical issues, privacy and model credibility was crucial for its wider application. Our survey results showed that 44.5% participants were concerned about the unclear attribution of responsibility in the event of AI misdiagnosis, and 17.1% participants were worried about data privacy and security issues. Who is responsible for the decisions made through the collaboration between physicians and AI tools? (33). Due to the “black box” nature of most AI algorithms, physicians find it difficult to understand how these algorithms generate their recommendations. Moreover, it remains unclear who should be held accountable and bear legal responsibility if harm is caused due to the use of AI tools by clinicians. Maintaining patient data security and privacy is of utmost importance. However, there is currently no comprehensive privacy protection and regulatory framework for AI in healthcare applications, which is why many participants are concerned about data privacy and security issues.

Our survey results showed that the main characteristics of the 21.1% participants who were unaware of the application of AI in healthcare were: older age, lower educational level, and living in rural areas. Individuals in this group are located in less information-developed regions and have a poorer ability to understand and accept new things. Due to concerns arising from a lack of information, they may be more likely to disagree with the application of AI in pediatric healthcare. Therefore, when promoting the application of AI in pediatric healthcare, special consideration needs to be given to these groups.

There were several limitations of this study. (1) This was a cross-sectional study with small sample size. (2) The data were collected through *ad hoc* questionnaires, and some participants may not have fully understood the questions posed, potentially leading to biases. (3) To our knowledge, there has been no previous evidence on this topic. This study was a exploratory study; therefore, we cannot assess the reliability and validity of the *ad hoc* questionnaire. (4) The survey was conducted in a hospital outpatient setting. The primary caregivers bringing children to the hospital for medical visits were mothers, followed by fathers. As a result, the majority of participants in this study were female (71.4%) and within the age range of 20–40 years. This introduced potential biases related to gender and age.

## Conclusion

This study explored parents’ awareness and attitudes toward the application of AI in pediatric healthcare through a questionnaire survey conducted among families of children. The findings indicate that the majority of respondents were aware of the application of AI in healthcare, and most parents recognize its convenience and efficiency. However, significant concerns remained regarding its accuracy, responsibility allocation, and data privacy and security. Additionally, the study revealed that parents from rural areas, with older age, and with lower levels of education had limited awareness of the application of AI in pediatric healthcare. These findings not only revealed the complex attitudes of parents toward the application of AI in pediatric healthcare but also highlighted the weak links in the dissemination of knowledge.

The study filled the gap in research regarding parents’ attitudes toward the application of AI in pediatric healthcare and provided important references for practice. When promoting the application of AI technology in pediatric healthcare, it is crucial to pay attention to parents’ concerns and needs, especially for those groups with insufficient understanding. Education and publicity should be used to enhance their comprehension and trust in AI technology. It is also necessary to optimize AI technology to improve its accuracy in pediatric healthcare, as well as to establish mechanisms for responsibility allocation and data privacy protection.

## Data Availability

The data analyzed in this study is subject to the following licenses/restrictions: the datasets used and analyzed during the current study are available from the corresponding author on reasonable request. Requests to access these datasets should be directed to Gao-Zhong Feng, 15060102098@163.com.
